# Machine learning-based prediction of acute kidney injury after nephrectomy in patients with renal cell carcinoma

**DOI:** 10.1038/s41598-021-95019-1

**Published:** 2021-08-03

**Authors:** Yeonhee Lee, Jiwon Ryu, Min Woo Kang, Kyung Ha Seo, Jayoun Kim, Jungyo Suh, Yong Chul Kim, Dong Ki Kim, Kook-Hwan Oh, Kwon Wook Joo, Yon Su Kim, Chang Wook Jeong, Sang Chul Lee, Cheol Kwak, Sejoong Kim, Seung Seok Han

**Affiliations:** 1 Department of Internal Medicine, Seoul National University College of Medicine, 103 Daehakro, Jongno-gu, Seoul, 03080 South Korea; 2 Department of Internal Medicine, Uijeongbu Eulji Medical Center, Eulji University, Uijeongbu-si, Gyeonggi-do South Korea; 3 Department of Internal Medicine, Seoul National University Bundang Hospital, Seongnam-si, Gyeonggi-do South Korea; 4 Medical Research Collaborating Center, Seoul National University Hospital, Seoul, South Korea; 5 Department of Urology, Seoul National University College of Medicine, Seoul, South Korea; 6 Center for Artificial Intelligence in Healthcare, Seoul National University Bundang Hospital, Seongnam-si, Gyeonggi-do South Korea

**Keywords:** Acute kidney injury, Urology

## Abstract

The precise prediction of acute kidney injury (AKI) after nephrectomy for renal cell carcinoma (RCC) is an important issue because of its relationship with subsequent kidney dysfunction and high mortality. Herein we addressed whether machine learning (ML) algorithms could predict postoperative AKI risk better than conventional logistic regression (LR) models. A total of 4104 RCC patients who had undergone unilateral nephrectomy from January 2003 to December 2017 were reviewed. ML models such as support vector machine, random forest, extreme gradient boosting, and light gradient boosting machine (LightGBM) were developed, and their performance based on the area under the receiver operating characteristic curve, accuracy, and F1 score was compared with that of the LR-based scoring model. Postoperative AKI developed in 1167 patients (28.4%). All the ML models had higher performance index values than the LR-based scoring model. Among them, the LightGBM model had the highest value of 0.810 (0.783–0.837). The decision curve analysis demonstrated a greater net benefit of the ML models than the LR-based scoring model over all the ranges of threshold probabilities. The application of ML algorithms improves the predictability of AKI after nephrectomy for RCC, and these models perform better than conventional LR-based models.

## Introduction

Renal cell carcinoma (RCC) represents approximately 3% of cancers, and is the 3rd most common type of cancer in the genitourinary tract^1^. During the last two decades, there has been an annual increase of 2% in its incidence worldwide^2^. In particular, small RCCs with T1 stage account for more than half of the newly diagnosed cases^3^. The early detection of small RCCs can improve overall survival of patients by curative nephrectomy^4^. Along with this trend, the American and European guidelines recommend partial nephrectomy (PN) rather than radical nephrectomy (RN) for localized tumors in stage T1 as a curative approach^2,5^. Despite an increasing tendency in performing PN, RN is also carried out, particularly in patients with chronic kidney disease, because of the high complication rate, long operation time, and potential morbidities of PN compared to RN^6–8^. The worsening of postoperative renal function continues to be a great issue in patients who undergo nephrectomy for RCC because of their superior survival and large remnant functioning tissues.

The loss of normal kidney tissues after PN or RN may result in an inevitable decline in kidney function despite the compensation of remnants^9–11^. Compensatory hypertrophy and hyperfiltration of the remaining kidney occurs within hours after nephrectomy, and a subsequent decrease in glomerular filtration rates is transient or subclinical^12^. However, 2–54% of patients experience postoperative acute kidney injury (AKI), which is attributable to several factors, such as elderly age, male sex, preoperative chronic kidney disease, diabetes mellitus, and RN^13–20^. AKI after nephrectomy for RCC leaves sequelae in the remaining kidneys, which is a strong risk factor for irreversible kidney dysfunction^18–20^. Furthermore, there is increasing concern that the transition to chronic kidney disease after nephrectomy is associated with both all-cause^21,22^ and cancer-specific mortality^23^.

Although previous studies have focused on postoperative kidney function after nephrectomy in the short- or intermediate-to-long term^13,14,16–19^, few models for predicting postoperative AKI have been developed. Moreover, these studies included patients who underwent certain types of surgery (e.g. laparoscopic or robot-assisted laparoscopic) rather than all kinds of operations^15,20^. Preparing for AKI beforehand may not be easy because several conditions in addition to operative settings have interactive and complex effects on the risk. The heterogeneous features of patients may also make it difficult to accomplish precise prediction. A previous logistic regression (LR) model (e.g., the simple postoperative AKI risk [SPARK] index) has suitable performance in predicting the risk of postoperative AKI in noncardiac surgery, but its performance has not been validated in the urologic surgery^24^. To overcome these limitations, we aimed to apply several machine learning models in predicting AKI after nephrectomy for RCC, and compared their performance with that of conventional LR models.

## Methods

### Patient and study design

A total of 4659 patients who were diagnosed with RCC and thus had undergone unilateral PN or RN between January 2003 and December 2017 were retrospectively reviewed. Patients were excluded if they met any of the following criteria: less than 18 years old (n = 11); metastatic RCCs (clinical T stage = 4; N stage > 0; and M stage > 0) (n = 331); previous history of nephrectomy (n = 3); kidney transplant recipients (n = 13); staged nephrectomy due to bilateral RCCs (n = 6); congenital single kidney before surgery (n = 4); presence of postoperative complications requiring re-operation (n = 3); and incomplete laboratory information (n = 184). Accordingly, 4,104 patients were analyzed in the present study. The study was approved by the institutional review boards of Seoul National University Hospital (H-1904-005-1021) and Seoul National University Bundang Hospital (B-1905-538-404) and was conducted in accordance with the principle of the Declaration of Helsinki. The requirement to obtain informed consent from the patients was waived by the above two IRBs.

### Study variables

Patient demographics such as clinical and laboratory data were recorded. Preoperative and intraoperative data (such as age, sex, body mass index, smoking status, hypertension, diabetes mellitus, histories of myocardial infarction, stroke, peripheral vascular disease, chronic hepatitis B and C, and other cancers, medications of angiotensin-converting enzyme inhibitors and angiotensin receptor blockers, type of operation, total and ischemic time of operation, estimated amounts of blood loss, intraoperative transfusion) and tumor-specific data (such as tumor size and clinical T stage) were extracted from electronic medical records. Blood laboratory data, such as preoperative serum creatinine, blood urea nitrogen, albumin, and hemoglobin, were obtained. For serum creatinine, postoperative values were also obtained. The estimated glomerular filtration rate (eGFR) was calculated using the Chronic Kidney Disease Epidemiology Collaboration equation^25^. Proteinuria was defined as ≥ 1+ on a dipstick test.

The primary outcome was postoperative AKI, defined as an increase in serum creatinine level to ≥ 0.3 mg/dL within 48 h or ≥ 1.5 times baseline within 7 days after operation according to the Kidney Disease Improving Global Outcomes guideline^26^. If the serum creatinine decreased within the non-AKI range and was at least 0.3 mg/dL below the peak level, the cases were defined as recovered AKI^27^.

### Statistical analysis

All analyses were implemented using R software (version 3.6.3; R Foundation for Statistical Computing). Comparisons of baseline characteristics were performed with the Wilcoxon rank-sum test for continuous variables and the chi-square test for categorical variables. The patients were randomly assigned to training (70%) and testing (30%) datasets. Using the training dataset, we developed machine learning models such as support vector machine (SVM), random forest, extreme gradient boosting (XGBoost), and light gradient boosting machine (LightGBM) to predict the risk of AKI. As a reference model, we used multivariable LR analysis (herein termed the LR-scoring model). Variables with a *P* value of < 0.2 in the univariate model were adjusted with a stepwise fashion. The logistic coefficients were used as clinical scores by proportionally assigning points and rounding to the nearest integer. For another reference, we used the SPARK index which had been validated in patients undergoing noncardiac operations^24^. SVM constructs a hyperplane in a high-dimensional space, which can be used for classification. Random forest is an ensemble of decision trees created by using bootstrap samples of the training dataset and random selection in tree induction^28^. For the random forest model, we used a grid search strategy to identify the best combination of hyperparameters with the caret package. XGBoost is an ensemble approach with a gradient descent–boosted decision tree algorithm^29^. We selected a low learning rate (0.0001), interaction depth of 5, and a maximum of 3000 iterations. LightGBM is an improvement framework based on the gradient descent–boosted decision tree algorithm and is more powerful than the previous XGBoost with a fast training speed and less memory occupation^30^. To minimize potential overfitting in the above machine learning models, we used tenfold cross-validation and out-of-bag estimation during development.

The model performance was assessed with the area under the receiver operating characteristic curve (AUROC), accuracy, and F1 score in the testing dataset. To calculate the performance of the SPARK index, we used the best threshold point of the curve. The DeLong test was used to compare AUROCs^31^. The net benefit over a specified range of threshold probabilities in outcome was evaluated using decision curve analysis^32,33^. The Hosmer–Lemeshow test was used to assess calibration. Two-sided *P* values less than 0.05 were considered significant.

## Results

### Baseline characteristics of the patients

The mean age of the patients was 56 ± 13 years and 2855 (69.6%) were male. 443 patients (10.6%) had diabetes mellitus. The proportion of patients who underwent PN was 66.5%. The median ischemic time during PN was 21 min (interquartile range 16–28 min). Postoperative AKI developed in 1167 patients (28.4%) after nephrectomy (423 after PN [15.5%] and 744 after RN [54.1%]; 817 [28.4%] in the training dataset and 350 [28.4%] in the testing dataset). 41.6% of patients with postoperative AKI had fully recovered renal function at discharge. Other baseline characteristics are shown in Table 1. These baseline characteristics did not differ between the training and testing datasets.

**Table 1:** Baseline characteristics of the study patients.

Variables	Total (n = 4104)	Training dataset (n = 2873)	Testing dataset (n = 1231)	*P* value
Age (years)	55.8 ± 12.8	55.7 ± 12.8	56.0 ± 12.8	0.573
Male (%)	69.6	69.7	69.1	0.691
Body max index (kg/m^2^)	24.7 ± 3.3	24.6 ± 3.3	24.7 ± 3.2	0.668
Current smoking (%)	24.4	24.3	24.8	0.724
**Comorbidities (%)**				
Diabetes mellitus	10.6	10.8	10.0	0.446
Hypertension	25.0	25.2	24.9	0.835
History of myocardial infarction	0.5	0.5	0.4	0.726
History of stroke	1.5	1.3	2.1	0.049
Peripheral vascular disease	0.1	0.03	0.08	0.537
Congestive heart failure	0.3	0.2	0.5	0.203
Hepatitis	4.4	4.5	4.1	0.585
History of cancer	4.5	4.3	5.0	0.309
Chronic obstructive pulmonary disease	1.6	1.9	1.0	0.035
ACEI or ARB (%)	8.4	8.3	8.7	0.693
Radical nephrectomy (%)	33.5	33.4	33.7	0.853
**Type of operation (%)**				0.425
Open	54.8	54.2	56.4	
Laparoscopic	12.6	13.1	11.5	
HALS	2.8	2.6	3.2	
Robotic	28.9	29.3	28.0	
LESS	0.9	0.8	0.9	
**Clinical T stage (%)**				0.692
T1	87.8	87.8	87.7	
T2	8.0	8.0	7.8	
T3	4.2	4.2	4.5	
Tumor size (cm)	3.2 (2.1–5.0)	3.2 (2.1–5.0)	3.2 (2.0–5.0)	0.744
Total operation time (min)	131 (100–174)	132 (100–170)	130 (100–175)	0.652
**Bleeding and transfusion amount**				
Estimated blood loss (mL)	150 (100–300)	150 (100–300)	150 (100–300)	0.602
Intraoperative RBC-transfusion (units)				0.687
0	96.0	95.8	96.4	
1–2	2.5	2.6	2.5	
3–5	0.9	0.9	0.7	
≥ 6	0.6	0.7	0.4	
**Preoperative laboratory findings**				
Hemoglobin (g/dL)	13.1 ± 1.7	13.1 ± 1.7	13.1 ± 1.7	0.665
Albumin (g/dL)	3.9 ± 0.6	3.9 ± 0.6	3.8 ± 0.6	0.055
Blood urea nitrogen (mg/dL)	14.1 ± 4.7	14.1 ± 4.8	13.9 ± 4.3	0.123
eGFR (mL/min/1.73 m^2^)	81.7 ± 18.6	81.9 ± 18.5	81.3 ± 19.0	0.342
Creatinine (mg/dL)	1.00 ± 0.28	0.98 ± 0.29	0.98 ± 0.26	0.928
**Proteinuria (%)**				0.017
0–trace	89.1	89.1	89.1	
1+	7.0	6.4	8.2	
2+	2.8	3.2	2.0	
≥ 3+	1.1	1.3	0.7	

*ACEI* angiotensin-converting enzyme inhibitor, *ARB* angiotensin II receptor blocker, *HALS* hand-assisted laparoscopic, *LESS* laparoendoscopic single-site surgery, *RBC* packed red blood cell, *eGFR* estimated glomerular filtration rate.

### Model performance in predicting AKI

When adjustment with a stepwise fashion was applied, several factors, such as male sex, diabetes mellitus, hypertension, RN, large tumor size, long operation time, intraoperative transfusion, and low eGFR were selected as risk factors for AKI in the LR-scoring model (Table S1). The corresponding clinical scores in this LR model are presented in Fig. S1.

**Figure 1:**
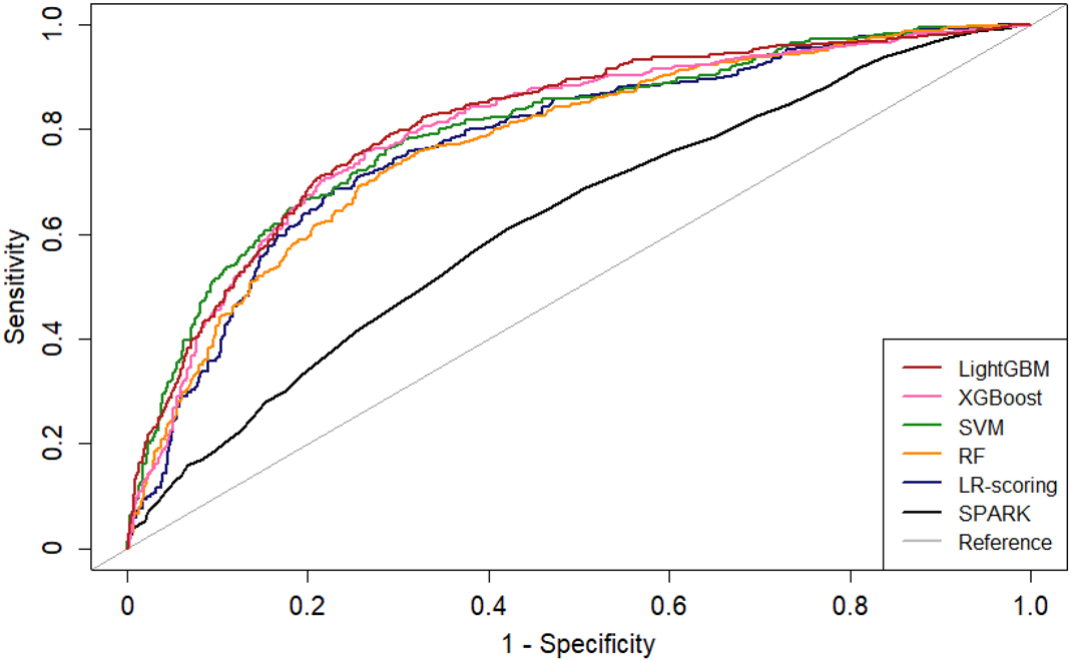
Receiver operating characteristic curves of the reference and machine learning models for predicting acute kidney injury after nephrectomy. *LightGBM* light gradient boosting machine, *XGBoost* extreme gradient boosting, *SVM* support vector machine, *RF* random forest, *LR* logistic regression, *SPARK* simple postoperative acute kidney injury risk.

**Figure 2:**
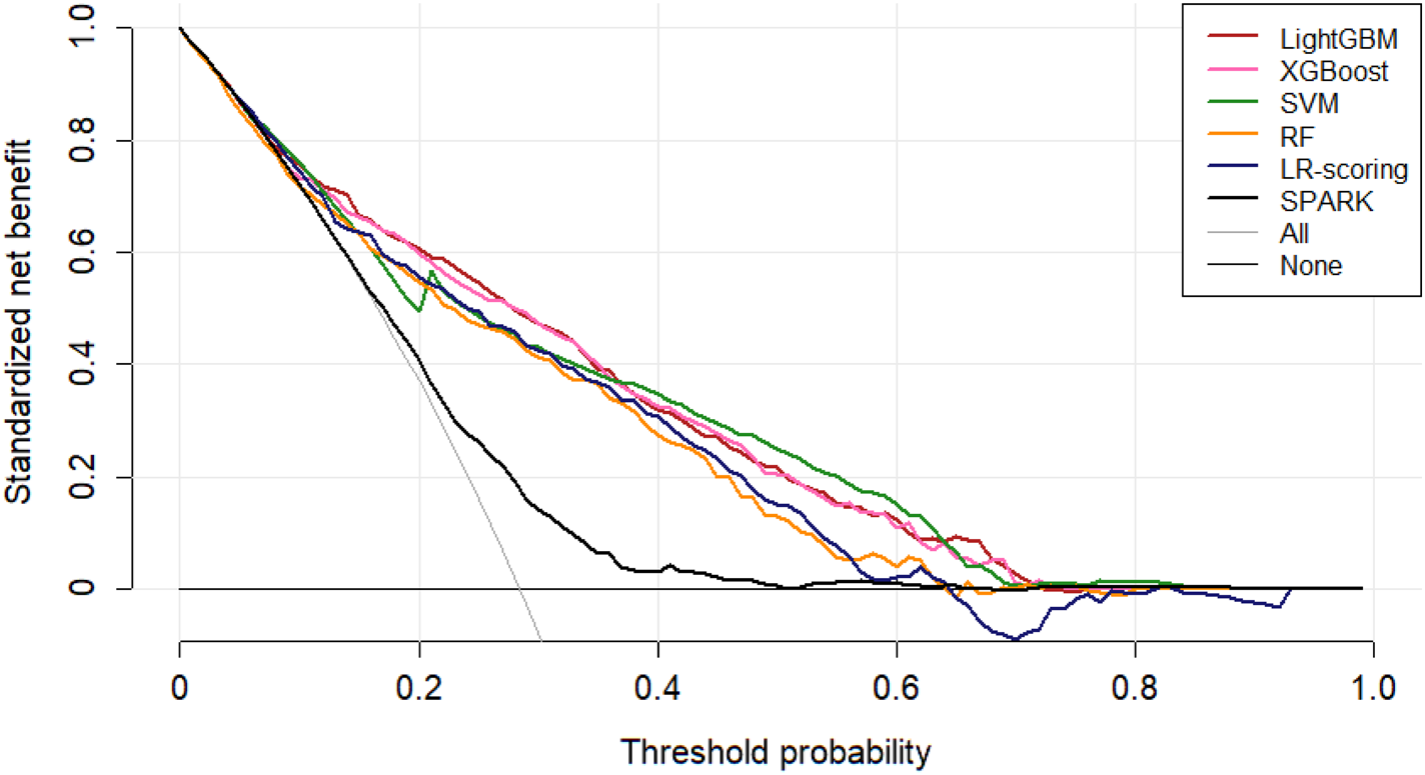
Decision curve analysis. *LightGBM* light gradient boosting machine, *SVM* support vector machine, *RF* random forest, *LR* logistic regression, *SPARK* simple postoperative acute kidney injury risk.

**Figure 3:**
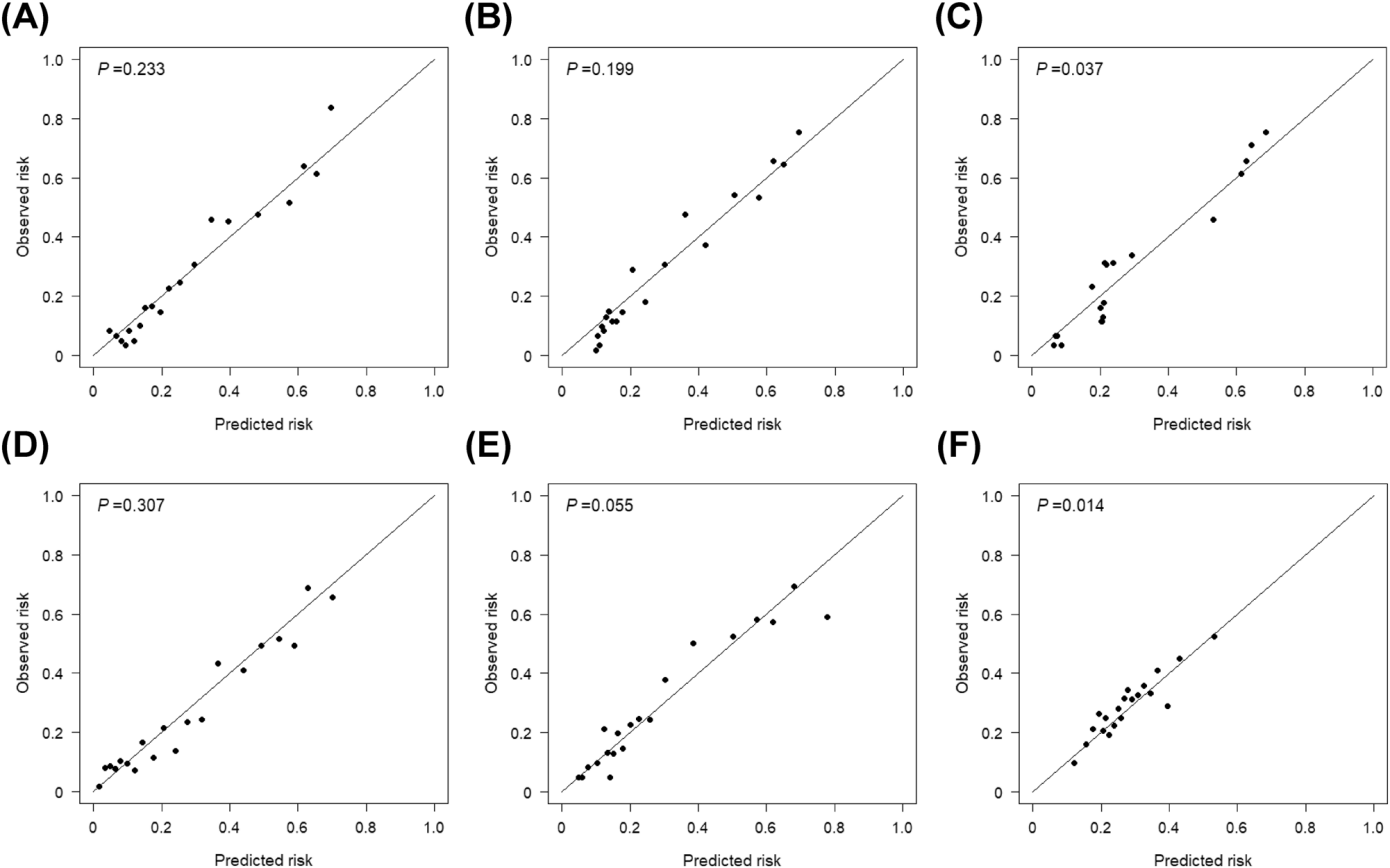
Calibration plots of the light gradient boosting machine (**A**), extreme gradient boosting (**B**), support vector machine (**C**), random forest (**D**), logistic regression-scoring (**E**), and simple postoperative acute kidney injury risk (**F**) models. *P* values more than 0.05 indicate good calibration.

We set up two LR-based models, the SPARK index and the LR-scoring model as a reference for comparison with the machine learning models. Among the models developed, the LightGBM model had the highest AUROC value (0.810 [0.783–0.837]), whereas the SPARK index showed the lowest AUROC value (0.626 [0.607–0.644]) (Table 2). All the machine learning models had higher AUROC values than the SPARK index. The LightGBM model had a higher AUROC value than the LR-scoring model with marginal significance. Corresponding curves supported these results (Fig. 1). When other performance indices, such as accuracy and F1 score, were examined, the XGBoost model had the best performance, and the LR-based models, including the SPARK index and the LR-scoring model, had the poorest performance. In decision curve analysis (Fig. 2), the net benefit was greater for machine learning models than for the SPARK index over all the ranges of threshold probabilities. The LightGBM, XGBoost and SVM models had the highest net benefits among the models. The LR-scoring model had a negative benefit in > 0.6 of the threshold probabilities. The LightGBM, XGBoost, random forest, and LR-scoring models were well calibrated (all *P* > 0.05), but the other models were not (all *P* < 0.05) (Fig. 3). Based on these results, the LightGBM model was chosen as the best model for predicting postoperative AKI.

**Table 2:** Comparison of models for predicting postoperative acute kidney injury after nephrectomy.

Model	AUROC (95% CI)	*P* value^a^	*P* value^b^	Accuracy (95% CI)	F1 score
SPARK index^c^	0.626 (0.607–0.644)	–	< 0.001	0.589 (0.574–0.604)	0.669
Logistic regression-scoring	0.775 (0.746–0.804)	< 0.001	–	0.759 (0.734–0.782)	0.837
Support vector machine	0.798 (0.770–0.826)	< 0.001	0.257	0.784 (0.760–0.807)	0.853
Random forest	0.791 (0.763–0.819)	< 0.001	0.447	0.771 (0.747–0.794)	0.851
XGBoost	0.807 (0.780–0.833)	< 0.001	0.144	0.786 (0.762–0.808)	0.856
LightGBM	0.810 (0.783–0.837)	< 0.001	0.084	0.777 (0.753–0.800)	0.851

*AUROC* area under the receiver operating characteristic curve, *CI* confidence interval, *SPARK* simple postoperative AKI risk, *XGBoost* extreme gradient boosting, *LightGBM* light gradient boosting machine.

^a^Compared with the SPARK index.

^b^Compared with the stepwise logistic regression model.

^c^The optimal cut off value was 29.5 in the present dataset.

### Variable ranking analysis

**Figure 4:**
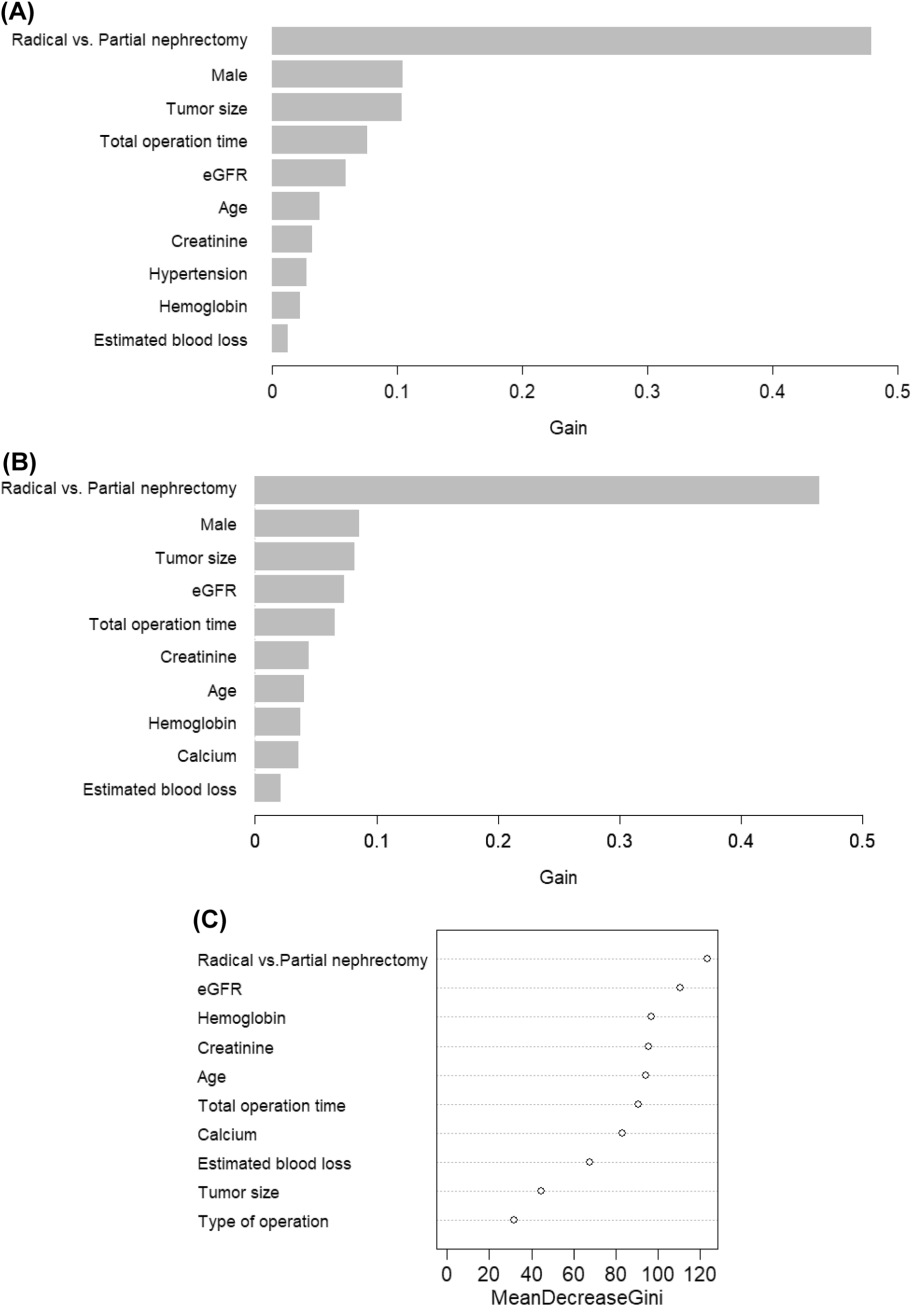
Importance of each variable in developing models such as light gradient boosting machine (**A**), extreme gradient boosting (**B**), and random forest (**C**) models. *eGFR* estimated glomerular filtration rate.

To estimate the contribution degree of each variable in predicting the risk of AKI, variable ranking analysis was performed (Fig. 4). Relative values ranged from 0 to 1, which indicated the proportional contribution of variables in predicting AKI. Accordingly, type of operation, sex, tumor size, operation time, and baseline eGFR were highly ranked as the top predictors.

## Discussion

It has become more important to precisely predict AKI in patients undergoing nephrectomy for RCC because surviving patients with AKI will suffer from subsequent chronic kidney disease and other worse outcomes. The present study first applied machine learning algorithms to accomplish the precise prediction of postoperative AKI, and the performance and calibration of these models were better than those of the LR-based reference models. Based on ranking analysis, certain variables were noted to contribute more to the predictive performance of the models. These results indicate that the precise prediction of postoperative AKI is achievable by machine learning despite the complex and interactive relationships of several variables.

A meta-analysis of 71 studies suggested that machine learning algorithms did not improve discriminative power over traditional LR-based models in predicting various clinical outcomes such as diabetes mellitus, infection, heart failure, and cancer^34^. Nevertheless, one study reported the superiority of machine learning models to the LR model in predicting AKI after minimally invasive laparoscopic or robot-assisted laparoscopic nephrectomy for RCC^15^. The present study dealing with all operation types supports this result with better model performance. Particularly, the performance improvement by the LightGBM model can be acceptable to alert clinicians of the risk of postoperative AKI.

Decision curve analysis takes into account the weights of different misclassification types with a direct clinical interpretation of the net benefit (i.e., the trade-off between undertreatment and overtreatment in the model)^32,33^. It is useful to compare models where the default strategies predict all-or-none outcomes such as AKI. All the machine learning models had greater net benefit over the range of threshold probabilities than the SPARK index. The LR-scoring model had a negative value of net benefit in a high range of threshold probabilities. These results provide clues on how machine learning models will be applicable to clinical practice.

The ranking analysis showed that certain variables such as nephrectomy type, patient characteristics (e.g., age and sex), and laboratory findings (e.g., eGFR and hemoglobin), contributed to the model performance. These results support the findings of previous large cohort studies focusing on postoperative AKI^14–19^. Only one or two variables may not be enough to accomplish a perfect prediction. Accordingly, modeling with at least the top variables obtained from the ranking analysis is needed if another model in an independent population should be developed.

Although the results were informative, some limitations should be discussed. The study design was retrospective in nature which may have potential selection bias. The study identified the most important variables with respect to predicting mortality, but we could not obtain certain degrees of risk, such as the relative risk, which is a common limitation of machine learning algorithms. The study results may not be applicable to some specific populations such as patients with metastasis or kidney transplant recipients. Concerns could be raised regarding other issues such as the absence of external validation and the effects of unidentified factors.

The application of machine learning algorithms improves the predictability of AKI after nephrectomy for RCC, and these models performed better than conventional LR-based models. If machine learning-based prediction models are successfully applied in clinical practice, the overall patient outcomes will improve by implementing earlier management. Future studies will explore whether machine learning is also applicable to predicting other outcomes after nephrectomy with validating results in independent cohorts.

## Supplementary Information

Supplementary Information.
