# Supplementary material for: Machine learning-based prediction of acute kidney injury after nephrectomy in patients with renal cell carcinoma
Source: Sci Rep. 2021 Aug 3;11:15704. doi: 10.1038/s41598-021-95019-1 (PMC8333365; doi:10.1038/s41598-021-95019-1)

**Machine learning-based prediction of acute kidney injury after nephrectomy in patients with renal cell carcinoma**

Yeonhee Lee^1,2^, Jiwon Ryu^3^, Min Woo Kang^1^, Kyung Ha Seo^4^, Jayoun Kim^4^, Jungyo Suh^5^, Yong Chul Kim^1^, Dong Ki Kim^1^, Kook-Hwan Oh^1^, Kwon Wook Joo^1^, Yon Su Kim^1^, Chang Wook Jeong^5^, Sang Chul Lee^5^, Cheol Kwak^5†^, Sejoong Kim^1,3,6†^, Seung Seok Han^1†^

^1^Department of Internal Medicine, Seoul National University College of Medicine, Seoul, Korea

^2^Department of Internal Medicine, Uijeongbu Eulji Medical Center, Eulji University, Gyeonggi-do, Korea

^3^Department of Internal Medicine, Seoul National University Bundang Hospital, Gyeonggi-do, Korea

^4^Medical Research Collaborating Center, Seoul National University Hospital, Seoul, Korea

^5^Department of Urology, Seoul National University College of Medicine, Seoul, Korea

^6^Center for artificial intelligence in healthcare, Seoul National University Bundang Hospital, Gyeonggi-do, Korea

^†^Co-corresponding authors

**Supplementary data table of contents**

Supplemental Table S1. Risk factors for acute kidney injury after nephrectomy in the logistic regression-scoring model

Supplemental Table S2. Simplified index in the logistic regression-scoring model

Supplemental Figure S1. Predicted risk of acute kidney injury according to the scores of the logistic regression-scoring model. AKI, acute kidney injury.

Supplemental Table S1. Risk factors for acute kidney injury after nephrectomy in the logistic regression-scoring model

|  | Coefficient (95% CI) | *P* value |
| --- | --- | --- |
| Male (vs. female) | 1.040 (0.815–1.266) | <0.001 |
| Diabetes mellitus (vs. none) | 0.333 (0.038–0.628) | 0.026 |
| Hypertension (vs. none) | 0.441 (0.220–0.661) | <0.001 |
| Radical nephrectomy (vs. partial) | 1.946 (1.721–2.171) | <0.001 |
| Tumor size |  |  |
| ≤4 cm | Reference |  |
| 4–7 cm | 0.287 (0.059–0.514) | 0.013 |
| 7–10 cm | 0.376 (–0.085–0.838) | 0.110 |
| ≥10 cm | 1.335 (0.664–2.007) | <0.001 |
| Total operation time (per 1 hour) | 0.165 (0.070–0.261) | <0.001 |
| Intraoperative transfusion (vs. none) | 0.475 (0.021–0.929) | 0.040 |
| Estimated glomerular filtration rate |  |  |
| ≥90 ml/min/1.73 m^2^ | Reference |  |
| ≥60 and <90 ml/min/1.73 m^2^ | 0.096 (–0.124–0.315) | 0.392 |
| ≥30 and <60 ml/min/1.73 m^2^ | 0.076 (–0.260–0.411) | 0.658 |
| <30 ml/min/1.73 m^2^ | 1.481 (0.237–2.726) | 0.019 |

Abbreviations: CI, confidence interval.

Supplemental Table S2. Simplified index in the logistic regression-scoring model

|  | Points |
| --- | --- |
| Male | 14 |
| Diabetes mellitus | 5 |
| Hypertension | 6 |
| Radical nephrectomy | 27 |
| Tumor size |  |
| ≤4 cm | 0 |
| 4–7 cm | 4 |
| 7–10 cm | 5 |
| ≥10 cm | 18 |
| Total operation time (per 1 hour) | ×2 |
| Intraoperative transfusion | 7 |
| Estimated glomerular filtration rate, <30 ml/min/1.73 m^2^ | 21 |

Supplemental Figure S1. Predicted risk of acute kidney injury according to the scores of the logistic regression-scoring model. AKI, acute kidney injury.


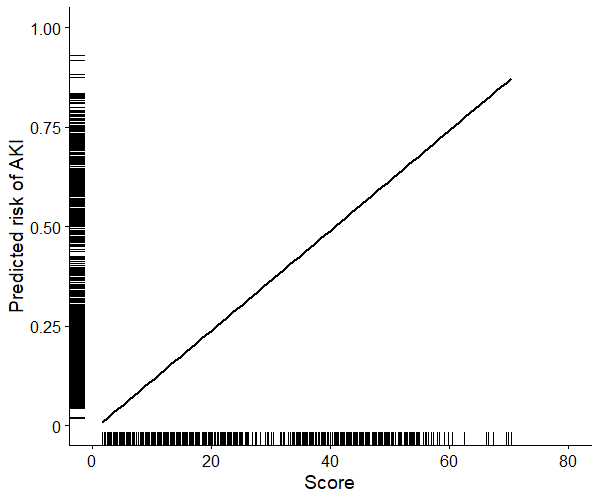

Supplement: Supplementary file 1 — Supplementary Information. [file 41598_2021_95019_MOESM1_ESM.docx]
